# One-year weight management lowers lipopolysaccharide-binding protein and its implication in metainflammation and liver fibrosis

**DOI:** 10.1371/journal.pone.0207882

**Published:** 2018-11-20

**Authors:** Hsiao-Ching Nien, Jin-Chuan Sheu, Yu-Chiao Chi, Chi-Ling Chen, Jia-Horng Kao, Wei-Shiung Yang

**Affiliations:** 1 Graduate Institute of Clinical Medicine, College of Medicine, National Taiwan University, Taipei, Taiwan; 2 Liver Disease Prevention and Treatment Research Foundation, Taipei, Taiwan; 3 Department of Family Medicine, National Taiwan University Hospital, Taipei, Taiwan; 4 Department of Internal Medicine, National Taiwan University Hospital, Taipei, Taiwan; 5 Graduate Institute of Epidemiology and Preventive Medicine, College of Public Health, National Taiwan University, Taipei, Taiwan; 6 Department of Surgery, National Taiwan University Hospital, Taipei, Taiwan; 7 Hepatitis Research Center, National Taiwan University Hospital, Taipei, Taiwan; Kaohsiung Medical University Chung Ho Memorial Hospital, TAIWAN

## Abstract

**Background:**

Studies showed that the endotoxemia-related biomarker, lipopolysaccharide-binding protein (LBP), is associated with obesity and fatty liver. The level of LBP is reduced after surgical weight loss. This study aimed to verify the change of serum LBP levels after one-year medical weight management in subjects with obesity.

**Methods and findings:**

A total of 62 subjects with obesity, 39 subjects with overweight, and 21 subjects with normal body mass index were enrolled for a one-year weight management program. Basic information, body composition analysis, clinical data, serum LBP level, and abdominal ultrasonography findings were collected. At baseline, the serum LBP levels of the obese and overweight subjects were significantly higher than that of the normal group (30.9±7.4 and 29.6±6.3 versus 23.1±5.6 μg/mL, respectively, *p*<0.001). Serum LBP in subjects with obesity was significantly reduced to 26.5±7.1 μg/mL (*p*-value < 0.001) after one year. In the multivariate analyses, LBP was associated with high sensitive C-reactive protein (hs-CRP) and non-alcoholic fatty liver disease (NAFLD) fibrosis score (NFS) before weight management in the obese group. Moreover, the change of LBP in response to weight management was significantly related to the changes of hs-CRP, leukocyte count and NFS by multivariate linear regression analysis also in the obese group.

**Conclusion:**

The serum level of the endotoxemia-related biomarker, LBP, decreases after one-year weight management in the obese subjects. In addition to serving as a metainflammatroy biomarker like hs-CRP, LBP may also be a potential biomarker as a non-invasive biomarker for the evaluation of liver fibrosis in NAFLD.

## Introduction

The prevalence of obesity increased significantly worldwide over the past few decades [1]. People with obesity also have an increased risk of comorbidities, such as non-alcoholic fatty liver disease (NAFLD), cancer, cardiovascular disease, type 2 diabetes mellitus (T2DM), hypertension, osteoarthritis, and stroke [2]. Body weight management with life style modification, pharmacotherapy, or bariatric surgery have been proven to improve some of these metabolic related diseases [3].

NAFLD is a disease involving hepatic fat accumulation and inflammation with the potential to progress from simple steatosis to non-alcoholic steatohepatitis (NASH), cirrhosis, and even to liver cancer [4]. It is well known to be highly associated with obesity, insulin resistance, and diabetes. The gold standard definition of NAFLD is fat acumination >5% in liver by histology without a history of significant alcohol consumption [4, 5]. Since current evidence does not support routine use of a liver biopsy in patients with suspected NAFLD or NASH [4], non-invasive tests such as liver enzyme levels, medical images, Fatty Liver Index, NAFLD fibrosis score (NFS), fibrosis-4 (FIB-4), and ultrasound methods are widely used as surrogate indicators [4, 6]. Abdominal ultrasound is a safe and convenient method for the diagnosis of NAFLD [4]. The sensitivity and specificity of abdominal ultrasound were reported as 94% and 84% for the diagnosis of NAFLD, and 57% and 88% in detecting liver fibrosis, respectively [7]. However, there is still no reliable serum biomarker representative of the severity of NAFLD.

Lipopolysaccharide-binding protein (LBP) is an acute-phase protein mainly derived from liver [8, 9]. LBP binds to the lipid A portion of lipopolysaccharide and interacts with toll-like receptor-4 (TLR-4)/ myeloid differentiation protein-2 (MD-2)/ cluster of differentiation 14 (CD14) protein complex to induce the downstream signaling pathways of innate immunity, such as nuclear factor-κB (NF-κB) and activator protein 1(AP-1), the major transcription factors involved in inflammation [10, 11]. The serum level of LBP increases with acute inflammation, especially in systemic infectious diseases [9]. It was demonstrated that the activation of TLR-4 signal pathway by lipopolysaccharide and LBP complex may lead to the progression of NAFLD from simple fatty liver to steatohepatitis and even further in animal models [11, 12]. Several human studies showed that serum LBP levels increased in NAFLD, NASH, hepatitis C virus (HCV) infection, obesity, insulin resistance, metabolic syndrome and atherosclerosis [8, 13–18]. These findings support that LBP could also be a biomarker indicative of effective endotoxemia in chronic low-grade inflammation related to cardio-metabolic diseases [8, 9, 13].

Moreover, serum LBP levels in patients with morbid obesity were reduced one-year after bariatric surgery [17]. In a 9-week diet intervention study enriching the specific gut microbiota such as *Bifidobacterium* spp, LBP was reduced as well as body mass index (BMI) [19]. The long-term effect of non-surgical weight management on serum level of LBP remains unclear. The aim of this study was to examine the impact of one-year weight management on serum LBP level in subjects with obesity and NAFLD versus normal and overweight subjects.

## Materials and methods

### Subjects

This is a cohort study with one-year follow-up. These 122 subjects visited the clinics were enrolled mainly for managing body weight due to metabolic diseases such as T2DM, hyperlipidemia, hypertension, metabolic syndrome, obesity, and fatty liver. Obesity was defined as BMI≧27 and overweight was defined as 24≦BMI<27 according to the guideline by the Department of Health and Welfare in Taiwan [20]. According to the baseline of BMI, the subjects were divided into three groups: 62 subjects with obesity, 39 subjects with overweight, and 21 subjects with normal BMI. Orlistat (81%), metformin (37%), and acarbose (50%) were prescribed to the subjects with obesity under doctors’ discretion for more than 3 months to control their body weight and T2DM. Moreover, the percentages of the subjects with overweight using metformin or acarbose were 24% and 33% and the subjects with normal BMI were 31% and 64%. Subjects with metabolic diseases received standard care of T2DM, hyperlipidemia or hypertension. Life style modification through diet and exercise education was prescribed to all participating subjects, even subjects with normal BMI.

No subjects had hepatitis B virus (HBV) infection, HCV infection, severe systemic infections, or alcohol addiction. Subjects with alcohol consumption over 21 drinks/week in men and 14 drinks/week in women were excluded [4]. All procedures involving human subjects were approved by the Research Ethics Committee of the National Taiwan University Hospital (Protocol ID No. 201407032RIFB). This study was carried out in accordance with the guidelines of the Declaration of Helsinki and the Institutional Review Board of National Taiwan University Hospital (Taipei, Taiwan). All the subjects gave written informed consent, and were regularly followed at least once every three months during one-year follow-up period.

### Basic information, body composition analysis, clinical data and abdominal ultrasound examination

Demographic data including age, gender, body weight, body height, BMI (weight in kilograms divided by height in meter squared), and waist circumference (WC, measured in cm at a level midway between the lowest rib and the iliac crest) were recorded. Body composition including soft lean mass, visceral fat mass, subcutaneous fat mass, and waist to hip ratio, etc., was analyzed by X scan plus II & ioi 353 (Jawon Medical co., LTD, Kyungsan-City, South Korea).

Standard clinical automatic analyzer was employed to assay the blood samples for fasting glucose, total cholesterol, triglycerides, high-density lipoprotein (HDL) cholesterol, low-density lipoprotein (LDL) cholesterol, uric acid, aspartate aminotransferase (AST), alanine aminotransferase (ALT), γ- glutamyl transpeptidase (γ-GT), high sensitive C-reactive protein (hs-CRP), and albumin with a Toshiba Automated Biochemical Analyzer C-8000 (Otawara-shi, Tochigi, Japan). Hepatitis B surface antigen (HBsAg), HCV antibody (anti-HCV), α-fetoprotein (AFP), insulin, thyroid-stimulating hormone (TSH), free thyroxine (free T4), cortisol, and C-peptide were assayed with a Abbott architect i2000 (Kallang Place, Singapore). Glycosylated hemoglobin (HbA1c) was analyzed with a Primus Ultra^2^ Variant Analyzer with Model 215 Auto-sampler (Kansas city, MO, USA). Leukocyte count (WBC) and platelet were assayed with a Sysmex Automated Hematology Analyzer X-1000 (Chuo-Ku, Kobe, Japan).

Serum LBP levels were measured using human LBP enzyme-linked immunosorbent assay kit (Biometec, Greifswald, Germany) as previously described [17, 18]. The degree of insulin resistance (IR) was calculated by the homeostatic model assessment (HOMA), using the formula: HOMA-IR = insulin (mIU/mL) x glucose (mg/dL)) x 0.055/22.5 [21].

NAFLD fibrosis score (NFS), which estimates the severity of liver fibrosis, were calculated based on Angulo’s formula using 7 variables = -1.675 + 0.037 x age (years) + 0.094 x BMI (kg/m^2^) + 1.13 x IFG (Impaired fasting glucose)/diabetes (yes = 1, no = 0) + 0.99 x AST/ALT ratio—0.013 x platelet (x 10^9^/l) -0.66 x albumin (g/dl) [22]. FIB-4 was calculated using the formula: age x AST / [platelet count (10^9^/L) x (ALT)^1/2^] as the index for liver fibrosis of different stages associated to cirrhosis [6].

The diagnosis of fatty liver was assessed by well-trained doctors using abdominal ultrasonography (Toshiba SSA-320A, SSA-660A, or Aplio 300, Otawara-shi, Tochigi-ken, Japan). The severity of fatty liver was divided into four grades: normal, mild, moderate and severe. The definition of mild fatty liver was an increased echogenicity of the liver compared with renal cortex. Severe fatty liver was defined when only the main portal vein walls could be visualized with absence of all smaller portal venule walls and/or gross discrepancy of the increased hepatic to renal cortical echogenicity. Moderate fatty liver was the intermediate between mild and severe fatty liver [23].

### Statistical analysis

SPSS Statistics software v19.0. was used for all statistical analyses. Continuous data were expressed as mean ± standard deviation and analyzed by the *t*-test. Paired *t* tests were used to compare the status of before and after weight management. The relationship between variables was also examined by simple correlation and backward multivariate linear regression. The variables with significant association were included in the multivariate linear regression models. Statistical significance is defined as *p*-value < 0.05.

## Results

There were 21 subjects with normal BMI, 39 with overweight, and 62 with obesity. At the baseline, the subjects with obesity had significantly higher body weight, BMI, WC, soft lean mass, visceral fat mass, waist to hip ratio, fasting glucose, insulin, HOMA-IR, triglycerides, uric acid, WBC, hs-CRP, ALT, γ-GT, C-peptide, and NFS than those in the normal BMI group (Table 1). The means of HDL cholesterol were higher in the normal BMI group. The subjects with overweight were similar to the subjects with obesity, except for fasting glucose, WBC, hs-CRP, ALT and γ-GT (Table 1).

**Table 1:** The characteristics of each group with different body mass index for one-year weight management.

BMI, Numbers of patients	Normal (<24, N = 21)			Overweight (24–27, N = 39)			Obesity (≧27, N = 62)		
	Before	After	*P-value*	Before	After	*P-value*	Before	After	*P-value*
**Age (years)**	48.0±10.0	49.0±10.0	-	49.6±10.0	50.6±10.0	-	47.9±10.8	48.9±10.8	-
**Gender (M/F)**	18/3		-	14/25		-	27/35		-
**Body weight (Kg)**	57.9±8.5	57.5±8.9	0.271	69.5±9.8	67.0±11.5	**< 0.001**	85.3±16.8	81.3±15.6	**< 0.001**
**BMI (kg/m**^**2**^**)**	21.8±1.9	21.6±1.9	0.286	25.9±0.7	24.9±1.6	**< 0.001**	30.9±4.4	29.4±4.1	**< 0.001**
**WC (cm)**	79.4±8.5	76.7±9.7	**0.027**	89.6±6.5	86.6±8.3	**0.001**	100.9±11.9	94.9±9.8	**< 0.001**
**Soft Lean Mass (Kg)**	39.4±6.0	39.0±6.1	**0.029**	44.3±8.9	43.8±9.2	**0.045**	52.2±12.2	51.2±11.0	**0.012**
**Visceral Fat Mass (Kg)**	1.6±0.5	1.6±0.6	0.819	2.8±0.5	2.5±0.8	**< 0.001**	4.3±1.3	3.7±1.4	**< 0.001**
**Subcutaneous Fat Mass (Kg)**	13.5±3.1	13.5±2.8	0.963	18.3±1.8	16.8±2.3	**< 0.001**	23.9±5.0	21.8±5.3	**< 0.001**
**Waist to Hip Ratio**	0.80±0.06	0.80±0.06	0.540	0.87±0.05	0.87±0.6	0.132	0.91±0.05	0.90±0.07	**0.001**
**Fasting glucose (mg/dL).**	92.7±7.7	96.2±10.8	0.104	98.0±17.0	95.7±12.4	0.332	99.7±12.3	98.5±14.7	0.856
**Insulin (mIU/mL)**	6.3±1.9	7.0±2.8	0.264	9.2±4.1	7.7±3.7	**0.015**	12.7±6.5	11.7±5.9	0.187
**HbA1c (%)**	5.5±0.4	5.8±0.5	**< 0.001**	5.6±0.7	5.8±0.6	**0.033**	5.7±0.5	5.8±0.5	**0.018**
**HOMA-IR**	1.4±0.5	1.7±0.7	0.148	2.3±1.3	1.9±1.3	**0.026**	3.1±1.9	2.9±1.8	0.344
**Total cholesterol (mg/dL)**	191.0±23.6	208.4±27.3	**0.049**	207.3±42.7	206.2±35.5	0.841	186.4±28.8	190.0±31.8	0.378
**Triglycerides (mg/dL)**	84.7±34.8	95.1±48.3	0.240	164.9±110.0	145.7±99.6	0.207	144.7±69.8	144.0±74.5	0.934
**HDL cholesterol (mg/dL)**	61.4±10.6	62.8±19.1	0.717	50.1±8.9	52.9±11.3	**0.042**	44.1±8.6	46.6±10.3	**0.006**
**LDL cholesterol (mg/dL)**	109.2±27.2	120.9±30.8	0.146	128.4±42.0	128.0±32.2	0.929	119.8±29.6	120.8±31.4	0.820
**Uric acid (mg/dL)**	5.0±1.1	4.8±1.4	0.117	5.9±1.5	5.7±1.4	0.147	6.2±1.6	5.8±1.5	**< 0.001**
**Leukocyte count**	5692±1562	6043±1714	0.203	6377±1339	6332±1272	0.814	6974±2019	6528±1508	**0.012**
**hs-CRP (mg/dL)**	0.01±0.05	<0.01	0.235	0.07±0.18	0.05±1.34	0.462	0.14±0.27	0.08±0.23	0.057
**Platelet (10**^**9**^**/L)**	278±47	287±70	0.539	277±56	264±50	**0.015**	279±68	276±67	0.623
**Albumin (g/dL)**	4.4±0.2	4.4±0.2	0.684	4.3±0.2	4.3±0.2	0.299	4.4±0.2	4.3±0.2	**0.022**
**AST (U/L)**	22.8±8.4	23.3±7.6	0.785	26.9±9.1	25.8±10.3	0.427	27.9±11.0	26.7±15.2	0.534
**ALT (U/L)**	25.1±21.7	22.1±10.5	0.405	34.9±23.0	34.4±33.4	0.904	43.3±26.7	39.0±29.8	0.282
γ**-GT (U/L)**	20.2±24.2	19.1±11.7	0.747	31.4±24.0	31.5±31.5	0.947	35.7±21.9	35.7±25.6	0.988
**TSH (μU/mL)**	1.44±0.65	1.36±0.78	0.633	1.90±1.01	2.01±0.91	0.397	1.92±1.23	1.89±1.31	0.831
**Free T4 (ng/dL)**	1.02±0.11	1.05±0.13	0.213	1.06±0.10	1.04±0.09	0.057	1.05±0.14	1.02±0.12	0.116
**Cortisol (μg/dL)**	9.14±3.86	8.57±4.03	0.579	9.5±4.0	9.8±3.5	0.668	9.39±3.38	8.69±2.58	0.128
**C-peptide (ng/mL)**	1.30±0.27	1.37±0.48	0.395	1.88±0.61	1.65±0.57	**0.008**	2.41±0.81	2.35±0.76	0.452
**NFS**	-2.795±1.042	-2.807±1.243	0.956	-2.034±0.946	-2.251±1.157	0.084	-1.870±1.401	-2.064±1.376	0.089
**FIB-4**	0.859±0.305	0.927±0.397	0.277	0.898±0.352	0.956±0.389	0.080	0.827±0.393	0.850±0.410	0.562
**Fatty liver**									
**Normal/Mild/Moderate/Severe**	9/12/0/0	9/11/1/0	-	5/18/10/6	3/27/4/5	-	1/15/20/26	5/17/16/24	-

Abbreviations: M/F, Male/Female; BMI, body mass index; WC, waist circumference; HbA1c, glycosylated hemoglobin; HOMA-IR, homeostatic model assessment for insulin resistance; HDL, high-density lipoprotein; hs-CRP, high sensitive C-reactive protein; AST, aspartate transaminase; ALT, alanine transaminase; γ-GT, γ-glutamyl transpeptidase; TSH, thyroid-stimulating hormone; NFS, Non-alcoholic fatty liver disease Fibrosis Score; FIB-4, fibrosis-4 index

The clinical characteristics of the subjects before and after one-year weight management are shown in Table 1. After one year, the average weight loss was 4.1±5.7 Kg (4.68 ± 5.85%) in the subjects with obesity. In the subjects with obesity, body weight, BMI, WC, soft lean mass, visceral fat mass, subcutaneous fat mass, waist to hip ratio, uric acid and WBC (Table 1) were significantly reduced, while HDL cholesterol and HbA1c were elevated. However, the levels of HOMA-IR, triglycerides, ALT, the severity of fatty liver, NFS and FIB-4 were not significantly different. The levels of hs-CRP and NFS tended to decrease (*p* = 0.057 and 0.089, respectively; Table 1). Moreover, the variables like BMI, WC, visceral fat mass, waist to hip ratio and HOMA-IR of the subjects with obesity still remained higher than the other two groups after the one-year weight management (*p*<0.05 for all). On the other hand, body weight, BMI, WC, soft lean mass, visceral fat mass, subcutaneous fat mass, insulin, HOMA-IR, C-peptide and platelet were significantly reduced, while HDL cholesterol and HbA1c were elevated in the overweight group. The HbA1c were significantly increased after one-year weight management in the subjects with normal BMI. This appears to be caused by two patients with poor DM control whose HbA1c were increased more than 10% of baseline values after one-year follow-up. However, the increase of HbA1c in the other groups were not statistically significant. For the normal BMI group, only WC and soft lean mass were significantly improved.

**Fig 1:**
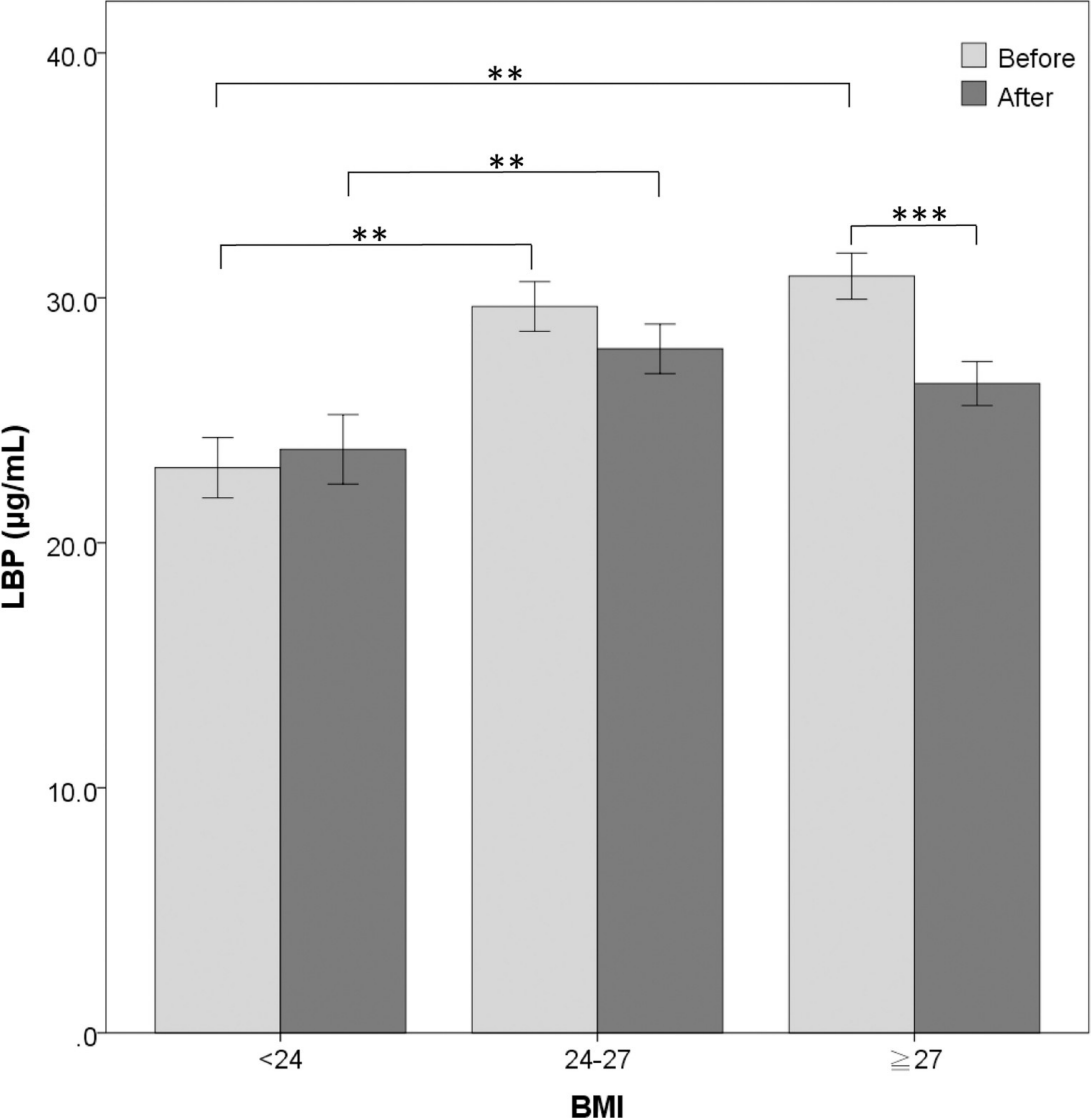
The serum levels of lipopolysaccharide-binding protein in each group with different body mass index before and after one-year weight management. Abbreviations: BMI, body mass index; LBP, lipopolysaccharide-binding protein. *** Comparison of the LBP levels before and after weight management with paired *t* test, *p*-value <0.001. ** Comparison between different BMI groups with *t* test, *p*-value <0.001.

Before weight management, baseline LBP levels were significantly higher in the subjects with obesity and overweight than that in the normal BMI group, respectively (30.9±7.4, 29.6±6.3 and 23.1±5.6 μg/mL, Fig 1). After one-year weight management, serum LBP level was significantly reduced by approximately 14% in the subjects with obesity (30.9±7.4 to 26.5±7.1 μg/mL; *p*<0.001, Fig 1), which was comparable to that of the overweight and normal BMI group, respectively (27.9±6.3 and 23.8±6.5 μg/mL, *p*>0.1; Fig 1).

The variables with significant changes after one-year weight management of the obese subjects were included as independent variables in the multivariate linear regression analyses by using serum LBP as the dependent variable (Table 2). In Table 2, the levels of LBP are significantly associated with hs-CRP in the subjects with obesity and overweight. NFS was only significantly associated with serum LBP level in the subjects with obesity before weight management but not after (Table 2). The serum level of LBP was not associated with any independent variable in the normal group.

**Table 2:** Multivariate linear regression analyses with circulating lipopolysaccharide-binding protein as the dependent variable.

BMI	<24 (N = 21)		24–27 (N = 39)		≧27 (N = 62)			
	Before		Before		Before		After	
	β	*p*-value	β	*p*-value	β	*p*-value	β	*p*-value
**Gender (M/F)**	8.127	0.221	-4.816	**0.030**	0.376	0.873	2.332	0.338
**hs-CRP (mg/dL)**	65.229	0.052	17.843	**0.002**	12.700	**<0.001**	9.856	**0.018**
**Leukocyte count (x1000)**	2.306	0.053	-0.870	0.283	0.444	0.384	1.065	0.115
**Triglycerides (mg/dL)**	-0.021	0.668	0.024	**0.041**	0.023	0.194	-0.010	0.477
**HDL cholesterol (mg/dL)**	0.213	0.178	0.133	0.372	0.097	0.515	-0.067	0.528
**Uric acid (mg/dL)**	-0.955	0.612	1.025	0.177	-0.412	0.537	-0.714	0.332
**NFS**	0.385	0.802	-0.703	0.533	1.638	**0.025**	0.682	0.310
**Fatty liver**								
**Mild vs. Normal**	-1.059	0.731	0.300	0.925	-11.807	0.111	-0.274	0.937
**Moderate vs. Normal**	-	-	1.071	0.744	-9.659	0.180	2.954	0.431
**Severe vs. Normal**	-	-	-0.953	0.804	-9.659	0.205	2.221	0.555
**Intercept**	3.145	0.844	17.269	0.117	33.641	0.003	26.290	0.003
**Adjusted R**^**2**^	0.159		0.231		0.268		0.121	

Abbreviations: LBP, lipopolysaccharide-binding protein; hs-CRP, high sensitive C-reactive protein; HDL, high-density lipoprotein; NFS, Non-alcoholic fatty liver disease Fibrosis Score. Statistically significant *p*-values are indicated in bold.

Consistent with a significant association between serum LBP and hs-CRP levels in the obese subjects, the change of serum LBP in these subjects correlated with changes of hs-CRP levels by correlation analysis (Table 3). The backward multivariate linear regression analysis revealed that the change of LBP level was significantly associated with the changes of hs-CRP, WBC, and NFS. The associations remained unchanged after the adjustment of the use of medications, such as orlistat, metformin and acarbose in the models of Table 3.

**Table 3:** Correlation and multivariate linear regression analysis between the change of lipopolysaccharide-binding protein concentration and the change of the indicated variables with one-year weight management.

Number of subjects	Obesity (BMI≧27), N = 62			
	r*	*p*-value*	*B*^#^	*p*-value^#^
Δ **hs-CRP (mg/dL)**	0.576	**<0.001**	17.106	**<0.001**
Δ **Leukocyte count**	0.224	0.081	1.199	**0.027**
Δ **Triglycerides (mg/dL)**	-0.209	0.103	-	-
Δ **HDL cholesterol (mg/dL)**	0.017	0.893	-	-
Δ **Uric acid (mg/dL)**	0.061	0.638	-	-
Δ **NFS**	0.162	0.209	1.874	**0.025**
Δ **Fatty liver**	0.084	0.515	-	-
**Intercept**	-	-	-2.557	0.001
**Adjusted R**^**2**^	-		0.384	

Abbreviations: LBP, lipopolysaccharide-binding protein; hs-CRP, high sensitive C-reactive protein; HDL, high-density lipoprotein; NFS, Non-alcoholic fatty liver disease Fibrosis Score. Statistically significant *p*-values are indicated in bold.

* Correlation analysis.

^**#**^ Multivariate linear regression analysis.

## Discussion

In this study, the subjects with obesity showed an average of 4.68% reduction in body weight after one year of weight management and the serum level of the endotoxemia-related biomarker LBP was significantly reduced by 14%. Nevertheless, other inflammatory and metabolic indicators, including fasting glucose, insulin, HOMA-IR, AST, ALT, γ-GT, C-peptide, and FIB-4, did not change as much as LBP.

Body weight reduction have been proven as a standard treatment of metabolic diseases [3]. Endotoxemia related biomarker, serum LBP, was also reduced by dramatic body weight loss after surgical intervention [17]. Moreover, LBP is an acute-phase protein [8, 9] that binds to lipopolysaccharide to induce the downstream TLR-4 signaling pathways of innate immunity and the inflammatory pathway of NASH, liver fibrosis and metabolic related diseases [10, 11, 15, 16]. The most important finding of this study was the association between LBP and NFS after one-year weight management without surgical interventions, which was rarely reported previously.

The American Association for the Study of Liver Disease (AASLD) guidelines recommends NFS as a non-invasive test for the evaluation of liver fibrosis in NAFLD [4]. NFS is known as a quantitative estimate of liver fibrosis in NAFLD. Serum LBP was shown to associate with hs-CRP, NALFD, and NAFLD with liver fibrosis [17, 24, 25]. Various studies have reported that losing weight in people with obesity reduced chronic low-grade inflammation, and improved NAFLD, NASH, NFS, T2DM, insulin resistance, cardiovascular diseases, and even cancer risk [17, 26, 27]. Our study showed that, for the subjects with obesity, the change in serum LBP levels correlated with the change in NFS in response to the one-year weight management.

Although the prevalence of NAFLD was estimated to be up to 30% among adults in industrialized countries, only a small number of NAFLD cases progress with liver inflammation that is generally known to be the second hit of the ‘two-hits’ hypothesis [28]. A convenient and non-invasive biomarker to predict the progression of liver fibrosis in NAFLD is an unmet clinical need. In our study, we observed a relationship between LBP and NFS in people with obesity, suggesting LBP might serve as a surrogate quantitative marker of fibrosis progression in NAFLD patients. This interesting and important issue needs further investigations.

Actually, we did not find the significant association in the overweight group or normal BMI group. The reason might be the percentages of severe and moderate fatty liver were low in non-obesity groups. Hence, the changes of NFS values were not significant in non-obesity group. Therefore, it was not easy to find the association between LBP and NFS in overweight group.

Although the reduction in serum hs-CRP not significant with weight loss in the subjects with obesity, we further showed that the change of LBP was highly correlated with the change of hs-CRP. The level of serum LBP was associated with hs-CRP both before and after one year of weight management. As hs-CRP is a widely accepted inflammatory marker, the results of the present study not only support the notion that weight loss has a general effect on reducing the inflammatory status in people with obesity [29] but also suggest that LBP may act similarly as hs-CRP as a biomarker.

However, there were some limitations of this study. First, there was no liver biopsy to evaluate the severity of fatty liver and liver fibrosis. Hence, we could not directly address the association between LBP and liver fibrosis. Second, the sample size was relatively small. Third, the effect on weight reduction was not tremendous. Only 37.1% of the obese subjects had more than 5% weight loss. Therefore, more intensive programs of weight loss are needed in the future. Fourth, it was difficult to evaluate the effect of metabolic medications on serum LBP in this study.

In summary, this study shows that one year of weight management significantly lowers the serum level of LBP in the obese subjects. A positive correlation is found between the change of serum LBP levels and the change in hs-CRP and NFS, implying LBP is not only a metainflammatroy biomarker, but might also be a potential biomarker like NFS as a non-invasive test for the evaluation of liver fibrosis in NAFLD.
